# Accelerated Thermal Cycling Test of Microencapsulated Paraffin Wax/Polyaniline Made by Simple Preparation Method for Solar Thermal Energy Storage

**DOI:** 10.3390/ma6051608

**Published:** 2013-04-29

**Authors:** Mahyar Silakhori, Mohammad Sajad Naghavi, Hendrik Simon Cornelis Metselaar, Teuku Meurah Indra Mahlia, Hadi Fauzi, Mohammad Mehrali

**Affiliations:** 1 Department of Mechanical Engineering and Advanced Material Research Center, University of Malaya, Kuala Lumpur 50603, Malaysia; E-Mails: msnaghavi@gmail.com (M.S.N.); hadidoank@gmail.com (H.F.); mohamad.mehrali@siswa.um.edu.my (M.M.); 2 Department of Mechanical Engineering, Universiti Tenaga Nasional, Kajang 43000, Selangor, Malaysia; E-Mail: indra@uniten.edu.my; 3 Department of Mechanical Engineering, Syiah Kuala University, Banda Aceh 23111, Indonesia

**Keywords:** Phase Change Materials (PCM), paraffin wax, energy storage, thermal reliability

## Abstract

Microencapsulated paraffin wax/polyaniline was prepared using a simple *in situ* polymerization technique, and its performance characteristics were investigated. Weight losses of samples were determined by Thermal Gravimetry Analysis (TGA). The microencapsulated samples with 23% and 49% paraffin showed less decomposition after 330 °C than with higher percentage of paraffin. These samples were then subjected to a thermal cycling test. Thermal properties of microencapsulated paraffin wax were evaluated by Differential Scanning Calorimeter (DSC). Structure stability and compatibility of core and coating materials were also tested by Fourier transform infrared spectrophotometer (FTIR), and the surface morphology of the samples are shown by Field Emission Scanning Electron Microscopy (FESEM). It has been found that the microencapsulated paraffin waxes show little change in the latent heat of fusion and melting temperature after one thousand thermal recycles. Besides, the chemical characteristics and structural profile remained constant after one thousand thermal cycling tests. Therefore, microencapsulated paraffin wax/polyaniline is a stable material that can be used for thermal energy storage systems.

## 1. Introduction

One of the most significant discussions in energy storage is the use of the Phase Change Materials (PCM) for thermal energy storage. PCMs are able to absorb and release large amounts of latent heat according to the increase and decrease in the temperature of the surroundings. They are classified as organic, inorganic and eutectic compounds [1,2,3]. Among PCMs, organic materials, such as paraffin and fatty acids, are more suitable for thermal energy storage, because of their high energy storage capability. Lifespan and the expenses regarding the use of storage material affect the economic feasibility of thermal storage system. Thence, significant changes in the melting point, as well as the latent heat fusion during the thermal cycling of phase change materials are not favorable. Commercial PCMs are used for latent heat energy storage, because of their availability and low cost [4,5,6,7]. A solar thermal system with latent heat storage undergoes one melt/freeze cycle per day. This might be called a normal cycle, while a repeated melt/freeze cycle test, conducted in the laboratory with a hot plate or similar system, is called an accelerated thermal cycle test [8]. Some issues regarding the use of these materials include the instability of material properties and corrosion of container [2]. The choice of PCM container is directly related to thermal stability of PCM material in such a way that it should be able to repeat the cooling and heating cycles. Sharma *et al.* have measured the melting point, latent heat of fusion and specific heat of stearic acid, acetamide and paraffin wax after cycling [9]. Paraffin wax and acetamide were found to be more stable over the 300 thermal cycles. However, the commercial grade of these materials has been measured after 1500 thermal cycles [10]. It has also been outlined that there is no obvious change of the melting point during thermal cycling. Thus, paraffin wax and acetamide have been considered to be promising PCM for some applications. Shukla *et al.* studied the thermal cycling of organic and inorganic PCM. Thereby, organic PCM has been considered more suitable than inorganic PCM for the purpose of thermal cycling tests [11]. On the other hand, the compatibility of PCM with other materials has attracted great attention of some researchers, because it directly affects the lifetime of encapsulation material, which covered the PCM. Some problems regarding the material compatibility with PCM have been explained by Mehling *et al.* [12], such as corrosion of the metal in contact with inorganic PCM, stability loss of plastics in contact with organic PCM and migration of liquid or gas through plastic, which in turn affect the performance of contained organic or inorganic PCM, as well as the outside environment. Thus, based on this feature, the PCM must have a long life during the thermal cycling test. Besides, the changes in latent heat values and phase transition temperature for a large number of melting and solidification processes must be as low as possible [13]. PCM should be tested by an accelerated cycle to measure the change in melting point, latent heat storage and specific heat, before being used in an actual thermal cycle. Uddin *et al.* studied the operation cycling of microencapsulated paraffin [14]. They evaluated the chemical structure, surface morphology and energy storage/release capacity after cycling test. Furthermore, Alkan *et al.* also carried out a number of investigations into the thermal reliability of microencapsulated docosane with polymethyl methacrylate (PMMA) [15]. They found that there is no significant change in latent heat and melting point after 1000, 3000 and 5000 cycles. In addition, Ahmet Sari *et al.* studied the accelerated thermal cycling test for microencapsulated n-octacosane for 1000, 3000 and 5000 repeated melting and freezing cycles [16]. They concluded that the chemical structures of microcapsules were not affected by thermal cycling. In another study, Sude Ma *et al.* conducted the thermal cycling test of microencapsulated paraffin wax/PMMA for 200, 500 and 1000 cycles [17]. They indicated that microencapsulated paraffin wax has satisfactory thermal reliability. 

In this study, we develop a facile method for the synthesis of paraffin wax/PAn microcapsules with different ratios of paraffin wax to polyaniline. Paraffin wax is used as latent heat storage material, and Polyaniline is used as the shell of this paraffin wax. The thermal reliability of the microencapsulated paraffin wax was distinguished by Thermal Gravimetry Analysis (TGA), Differential Scanning Calorimeter (DSC), Fourier transform infrared spectrophotometer (FTIR) and Field Emission Scanning Electron Microscopy (FESEM) devices.

## 2. Experimental Method

### 2.1. Materials and Method

Paraffin wax with a melting point of 53–57 °C and aniline (C_6_H_7_N) were used as core and shell materials, respectively. Ammonium persulfate (APS, (NH_4_)_2_S_2_O_8_) was used as the oxidant and silicon oil for a uniform heating rate during polymerization. All chemicals were used as received without further purification. Water purification was done through distillation followed by deionization with the aid of ion-exchange resins.

Paraffin wax/PAn microcapsules were synthesized via *in situ* polymerization of adsorbed aniline monomer on the surface of paraffin wax. In the first step, paraffin wax was melted in deionized water (150 mL) in a 300 mL beaker at 75 °C, and then aniline was added as a monomer to the beaker, followed by stringing for 1 h at the speed of 700 RPM in various ratios of Paraffin wax/PAn 0.1/0.9, 0.2/0.8, 0.3/0.7, 0.7/0.3 g, that is named S1, S2, S3, S4, respectively. The beaker should be kept in silicon oil for a uniform heating rate. In the second step, 2.28 g of APS was dissolved in deionized water (100 mL) in the 300 mL beaker at the same condition of the paraffin wax and was added drop-wise to the beaker. Finally, the reaction proceeds by the chemical oxidation of aniline monomers with APS. Moreover, the color of the solution started to change gradually from the first hours. Initially, it was green, and after 2 h, the color changed to red. For the third hour, the red color became darker. Ultimately, when the polymerization was completed, it became almost black.

### 2.2. Experimental Setup and Procedure

**Figure 1:**
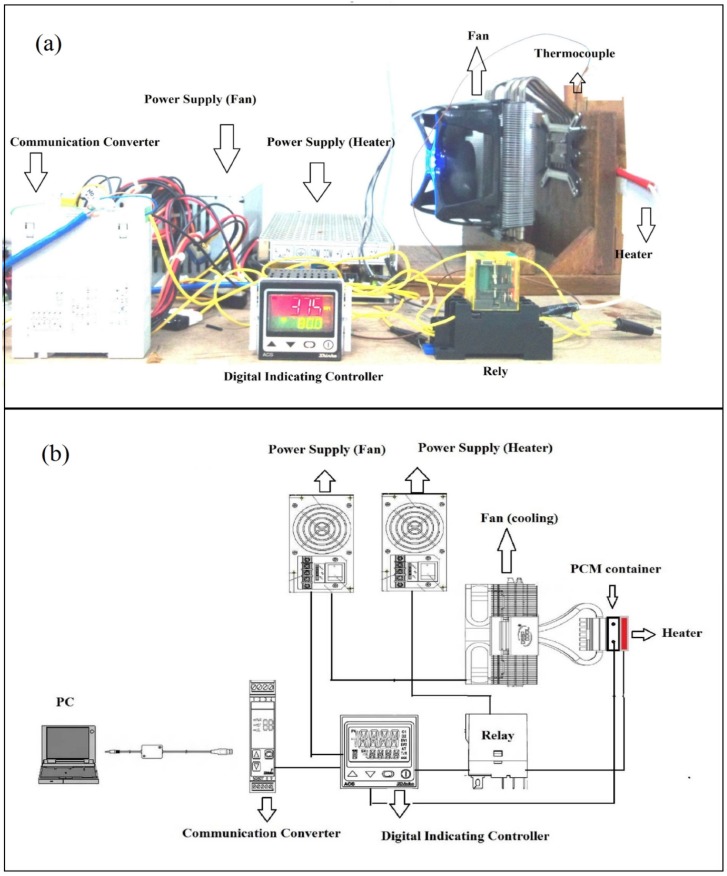
(**a**) Image of repeated thermal cycling test; (**b**) schematic of repeated thermal cycling test [18].

The experimental setup included a strip heater (24VDC), deep cooler (12VDC), PCM storage box, thermocouple, temperature controller (ACS-13A-R/M-Shinko), communication converter (IF-400-Shinko) and a PC for data acquisition system. The PCMs are kept in a rectangular box that is made of copper. The width length and height of the box are 12 mm, 6 mm and 35 mm, respectively. The box was filled with 1 g of PCM. The stainless steel strip heater was glued to the outside of the copper box and its flux voltage set to 80 W and 24 V, respectively. The deep cooler consisted of a heat pipe heat sink to cool the storage box. The copper surface of the cooler was attached on the other side of the storage box. K-Type thermocouples were used in this experiment, which has temperature range of 0–1260 °C and a limited error of ±0.7%. Figure 1 shows the image and schematic of the thermal cycling setup [18].

### 2.3. Characterization of Phase Change Material

A Differential Scanning Calorimeter (DSC) (model: METTLER TOLEDO 820C-Error ±0.5–1 °C) was used to analyze the thermodynamic characteristics of the microencapsulated paraffin wax. In this evaluation, DSC was used to measure latent heat, heat capacity and melting temperature of the samples. Moreover, the chemical structure stability of microencapsulated was examined using a Fourier transform infrared spectrophotometer (model: PerkinElmer Spectrum 400). With TGA (model: METTLER TOLEDO SDTA 851-Error ±5 µg ), the microencapsulated paraffin wax was generally heated at a constant rate of 10 °C per minute, and the resulting temperatures and the degradation rate were measured as a function of time or temperature. FESEM was used to study the morphology of the samples. Sample capsules were mounted on copper stubs with dark double-sided carbon tape and vacuum-coated with a platinum film (Ion Sputtering Device) and then examined by FESEM (model: Zeiss Auriqa).

## 3. Results and Discussions

### 3.1. Thermogravimetry Test 

**Figure 2:**
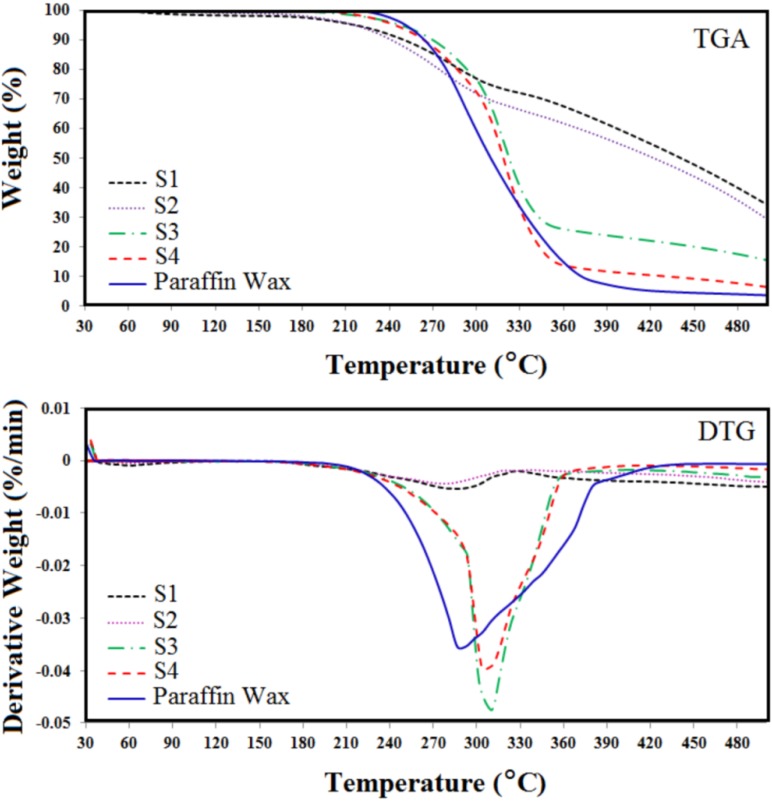
Thermal Gravimetry Analysis (TGA) and Differential Thermal Gravimetry (DTG) results of microencapsulated S1, S2, S3, S4 and paraffin wax.

Microencapsulated paraffin wax was subjected to heating and cooling cycling test. The TGA and Differential Thermal Gravimetry (DTG) result of the capsules are shown in Figure 2.

The TGA result shows the weight loss of microencapsulated paraffin wax in terms of temperature. The rates of weight loss are 55%, 60%, 75%, 80% and 100% for S1, S2, S3, S4 and paraffin wax, respectively. This can be explained by the fact that the weight loss of the microencapsulated paraffin wax depends on the encapsulation ratios of paraffin wax to the microencapsulation [19]. In other words, the rate of decomposition of the paraffin wax is decreased due to the increase in coating material. This implies that the shell structures of microencapsulated paraffin wax provided a better protection and prevented the paraffin wax from leaking out of the capsules. In addition, Differential Thermal Gravimetry (DTG) tests for the capsules show the rate of weight loss with temperature. As can be seen, the paraffin wax degrades in one step, while paraffin wax/polyaniline microcapsules degrade in two steps. The degradation of paraffin wax begins at around 280 °C, and the degradation of paraffin/Polyaniline is around 280 °C and 330 °C, with the second step belonging to polyaniline. This means that the degradation of the polyaniline is at higher temperature than that of paraffin wax. Therefore, polyaniline can protect the paraffin wax as a core material. The recent reports show that the decomposition temperature of polyaniline depended on the polymerization condition of aniline monomer [20]. From the figure, it is clear that encapsulated paraffin wax, S1 and S2, are more stable than S3 and S4, due to a better encapsulation. It means that the high ratio of polyaniline can cover most of the paraffin wax during polymerization. Although the latent heats of capsules S1 and S2 are expected to be less, due to the low content of paraffin wax, these two capsules could be applied in thermal energy storage systems with less failure and more longevity. Hence, microencapsulated paraffin wax, S1 and S2, can be chosen to be put in thermal cycling tests due to their high thermal stability.

### 3.2. Repeated Thermal Cycling Test 

**Figure 3:**
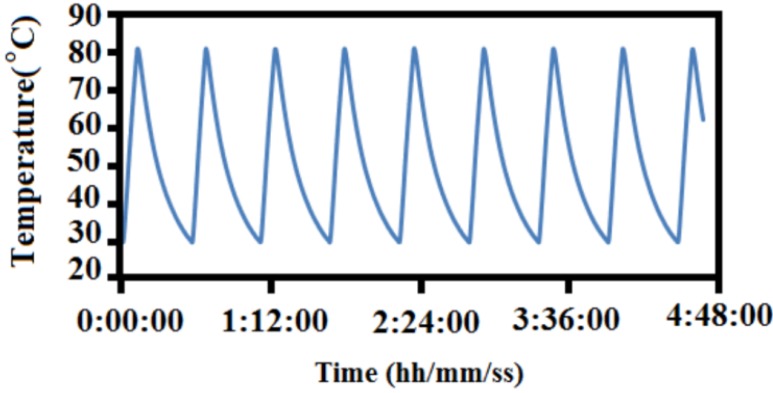
Thermal cycling operation of hot plate heating and cooling temperature with time.

Microencapsulated paraffin waxes were analyzed in the repeated cyclic state. The set-up is displayed in Figure 1. The history of temperature-time for a few cycles is shown in Figure 3 Microencapsulated paraffin wax was tested through 200, 400, 600, 800 and 1000 cycles.

### 3.3. Thermal Properties of Microencapsulated Phase Change Material (MEPCM)

**Figure 4:**
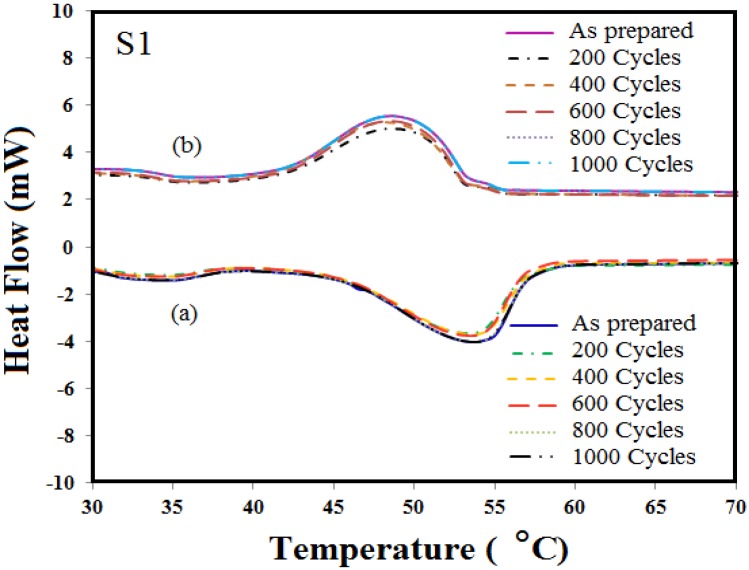
Differential Scanning Calorimeter (DSC) curves of different cycling tests for S1:(**a**) heating; (**b**) cooling.

**Figure 5:**
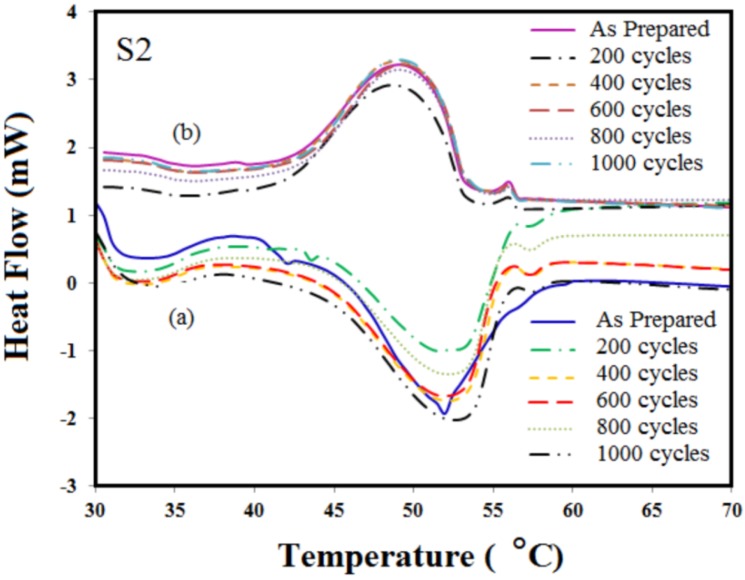
DSC curves of different cycling tests for S2: (**a**) heating; (**b**) cooling.

Figure 4 and Figure 5 show the DSC result of the microencapsulated paraffin wax, S1 and S2, after 200, 400, 600, 800 and 1000 cycles, respectively. DSC curves show the melting temperature of S1 and S2. The melting points of the capsules, S1, have changed 53.2–53.5 °C for the melting process and between 46.4 °C and 45.4 °C for the cooling process after repeated thermal cycles. The melting and solidifying temperature of S2 are around 53.4–53.8 °C and 44–46 °C, respectively. 

Based on these results, phase change temperatures of the capsules have a good thermal reliability during the cycling test. Congruent melting was distinguished by its constant phase change temperature. No supercooling was observed, no significant change in volume took place and material degradation was not seen. Table 1 and Table 2, show the latent heat of heating and freezing of capsules S1 and S2 after different recycles. The average change in the specific enthalpies of heating and cooling are around 30 to 32 J/g and 60 to 65 J/g for S1 and S2, respectively. Moreover, the latent heat of microencapsulated S1 has a variation of 1.2%, 3.2%, 2.2%, 3.2% and 1.6% for melting and 3.3%, 3.3%, 6.1%, 5.8% and 5.8% for the freezing process after 200 to 1000 cycles, respectively, compared with zero cycles. Besides, capsules S2 have shown variation of 4.6%, 6.2%, 6.2%, 6.2% and 7% during their melting process and 4.3%, 6.6%, 6.6%, 7.8% and 8.1% during their solidification process for the same range of cycles (200–1000). This might stem from the moisture content in the samples, as they absorb moisture from the surroundings, or the quality of core materials. This kind of variation is also observable in pure material [10]. It is clear that there is no significant change in the latent heat capacity and temperature of melting and freezing after thermal cycling, and increasing the number of cycling did not lead to any degradation or change in the chemical structure of the paraffin wax. Therefore, the heat storage material could form the first crystal structure (in a fresh state of PCM) during the solidification period of the repeated thermal cycling. Low impurity or having no impurity at all can be the cause of no degradation in the PCM during thermal cycling. The reason might be that there was no chemical reaction during thermal energy storage, as well as the release process within the material itself. Besides, no chemical reaction occurred in the coating material (polyaniline) or with the holding container. Both samples displayed their reproducibility in thermal performance and melting and solidifying behavior. They also exhibited an acceptable thermal reliability of the capsules being heated by the hotplate. They are able to guarantee a long-term performance of heat storage. 

**Table 1:** DSC result of the microencapsulated paraffin wax, S1.

Cycling number	Melting temperature (°C)	Melting latent heat (J/g)	Freezing temperature (ºC)	Freezing latent heat (J/g)
0	53.2	31.0	46.4	32.6
200	53.2	30.6	44.9	31.5
400	53.3	30.0	45.2	31.5
600	53.3	30.3	45.1	30.6
800	53.5	30.6	45.5	30.7
1000	53.4	30.5	45.4	30.7

**Table 2:** DSC result of the microencapsulated paraffin wax, S2.

Cycling number	Melting temperature (°C)	Melting latent heat (J/g)	Freezing temperature (ºC)	Freezing latent heat (J/g)
0	53.8	65.1	44.9	66.4
200	53.2	62.1	44	63.5
400	53.3	61	44.1	62
600	53.3	61	44	62
800	53.3	61	44.2	61.2
1000	53.4	60.5	46.1	61

Here, two important parameters used to analyze the phase change properties of paraffin wax (using DSC measurements) would be introduced. One is the encapsulation ratio (*R*) and the other is encapsulation efficiency (*E*) denoted by the following equations [21,22]:

(1) $$R=\frac{∆H_{m,MicroPCMs}}{∆H_{m,PCM}}\times 100\%$$

(2) $$E=\frac{∆H_{m,Micro-PCMs}+∆H_{c,Micro-PCMs}}{∆H_{m,PCMs}+∆H_{c,PCMs}}\times 100\%$$

where $∆H_{m,PCMs}$ and $∆H_{c,PCMs}$ indicate the fusion heat and crystallization enthalpy of the bulk paraffin wax, respectively; $∆H_{m,Micro-PCMs}$ and $∆H_{c,Micro-PCMs}$ designate the fusion heat and crystallization enthalpy of the microencapsulated one, respectively. Effective encapsulation for paraffin wax within the microcapsules is specified by encapsulation ratio, whereas the loading content (10% and 20% for S1 and S2, respectively) is determined by the dry weight percent of the core material. By this notation, the encapsulation ratio signifies the effective performance of paraffin wax inside capsules for heat energy storage and thermal regulation. Besides, both melting and crystallization enthalpies influence the value of encapsulation efficiency.

The encapsulation ratio and encapsulation efficiency of the microencapsulated paraffin wax obtained by DSC measurement, as well as calculations are manifested in Table 3. It can be noted that these two parameters are proportional to the paraffin wax/polyaniline weight ratio that dominates the core material loading. However, upon the synthesis of microcapsules S1 and S2, a compact shell is achieved that engenders an effective encapsulation, preventing any leakage from capsules. Moreover, the monomer polymerization was not performed completely, resulting in washing out of some of the monomers from the product. This led to a high encapsulation ratio and encapsulation efficiency for the samples, S1 and S2. 

**Table 3:** The phase change behavior and performance of microencapsulated paraffin wax.

Sample Name	$∆H_{m}(J/g)$	$∆H_{c}(J/g)$	Paraffin wax loading (%)	Encapsulation ratio (%)	Encapsulation efficiency (%)
Paraffin wax	131.92	132.31	–	–	–
S1	31	32.6	10	23.4	24.0
S2	65.1	66.4	20	49.3	49.7

### 3.4. Structure Stability of MEPCM

**Figure 6:**
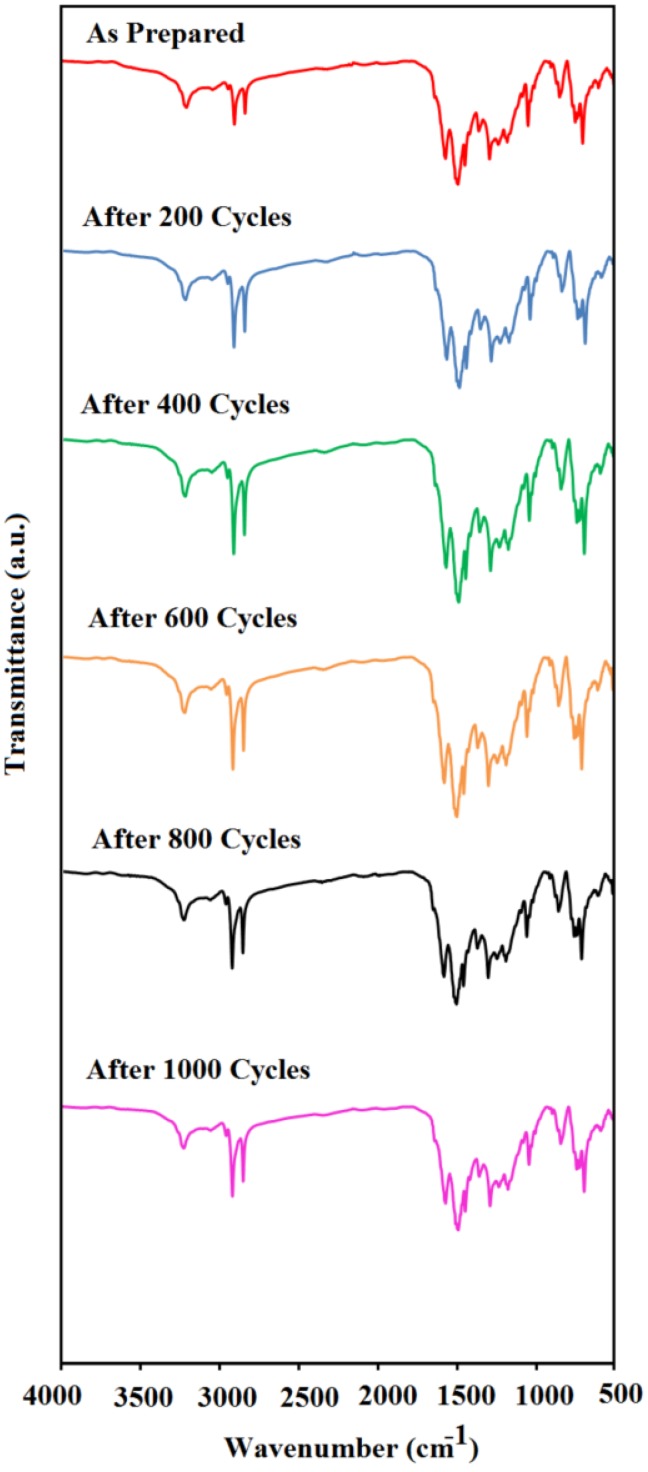
Structure stability of microencapsulated S1 after different cycling tests.

According to the FTIR result of Sample 1 (S1), the characteristic peaks of the paraffin wax and polyaniline can be observed after 200, 400, 600, 800 and 1,000 cycles (Figure 6). The peaks around 2960–2850 cm^−1^ and 1465 cm^−1^ show carbon hydrogen stretching and bending absorption, respectively. The symmetric C–H bending absorption of the CH_3_ group at 1381 cm^−1^ and the CH_2_ rocking absorption band at 729 cm^−1^ confirm the linear saturated aliphatic structure of the paraffin wax [23]. From the IR spectrum, six major absorptions can be observed: at 1592, 1503, 1307, 1220, 1155 and 824 cm^−1^, of which 1592 and 1503 cm^−1^ belong to stretching vibrations of C–C ring and the peaks at 1220 and 1370 cm^−1^ are related to N–H bending, as well as the C–N (or C–C) stretching. The peaks at 1155 and 824 cm^−1^ are also assigned to the in-plane and out-of-plane C–H bending modes. The bands of the polyaniline salt are also shown at 1498, 1462, 1306, 1274, 1074 and 789 cm^−1^. Besides, the spectrum for the polyaniline salt demonstrates peaks around 3264, 1653 and 634 cm^−1^. The band of 3264 cm^−1^ is assigned to the NH_2_ stretching mode and the 1653 cm^−1^ peak to the NH_2_ bending vibration. Finally, the 634 cm^−1^ is related to NH_2_ wagging. In addition, FTIR results shows that with increasing the number of cycles, the change in all peaks is negligible. Figure 6 also indicates that the characteristics of microencapsulated paraffin wax remain stable after 1000 cycles. It means that the compatibility of the coating material and core remain in good conditions after repeated thermal recycles. Moreover, the FTIR results do not show any new peak in comparison with the FTIR of S1 and S2 before the cycling test. Consequently, the results confirm that the reaction between encapsulation materials and the environment is not significant. The low tendency of polyaniline to react with paraffin wax and the high stability of polyaniline can also be considered as two strong reasons behind the ability of polyaniline as a suitable coating for encapsulation of paraffin wax.

### 3.5. Surface Morphology of MEPCM

**Figure 7:**
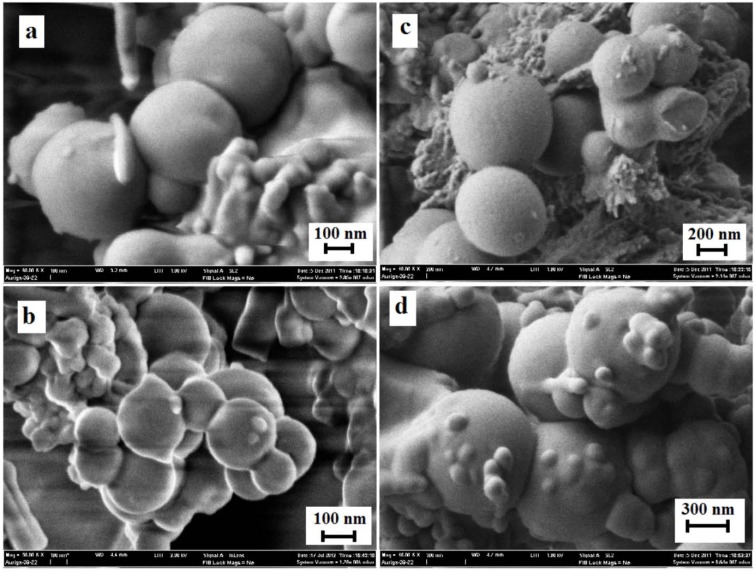
(**a**) Microcapsules S1 after 0 cycle; (**b**) microcapsules S1 after 1000 cycles; (**c**) microcapsules S2 after 0 cycle; and (**d**) microcapsules S2 after 1000 cycles.

Figure 7 shows the surface morphology of microcapsules S1, S2 after 0 and 1000 cycles. Physical properties of the capsules can be analyzed by their shape and size. In other words, the comparison of FESEM results for the microcapsules before and after cycling can show the stability, solubility and chemical reactivity of the sample. As noted from this figure, the paraffin wax was encapsulated by polyaniline. S1 and S2 formed globular capsules with average sizes within the range of 300 to 500 nm. This shape remained stable after 1000 cycles. The FESEM results confirm that the flexibility of polyaniline is acceptable, because no cracks were observed on the surface of the polyaniline coating after 1000 cycles. The results of DSC, FTIR and FESEM confirm that the polyaniline is a suitable coating for the purpose of microencapsulating paraffin wax. 

## 4. Conclusions

In a word, a facile method has been used for the preparation of microencapsulated paraffin wax/polyaniline by an *in situ* polymerization method. TGA results show that the thermal stability of the microencapsulated paraffin wax/polyaniline with the ratio of (1:9) and (2:8) is better than that of (3:7) and (7:3). To investigate the thermal reliability of the capsules, microencapsulated paraffin waxes, S1 and S2, were tested by thermal cycling. DSC results indicated that the average latent heats of melting and freezing of the microencapsulated paraffin wax/polyaniline were around 30–32 J/g and 60–65 J/g for S1 and S2, respectively. These imply that the microencapsulated paraffin wax/polyaniline were reliable in terms of the thermal cycling test. Furthermore, the FTIR spectroscopy results indicated that the accelerated thermal cycling does not cause any degradation in the chemical structure of the PCM. This means that the reaction between encapsulation materials and environment is not significant. FESEM analysis also showed that the microcapsules prepared by *in situ* polymerization were globular in shape. Besides, the surface morphologies of the capsules with the ratio of (1:9) and (2:8) are homogenous after 1000 cycling test. This indicated that the coating materials were suitable for encapsulation of PCM in thermal energy storage at high temperatures. Based on all these results, accelerated thermal cycling tests of microencapsulated paraffin wax/polyaniline reveal that the change in the melting and freezing temperatures have negligible magnitudes for latent thermal energy storage (LHTES) applications, and the Microencapsulated Phase Change Material (MEPCM) has good long-term reliability.
